# Extracellular carbonic anhydrase: Method development and its application to natural seawater

**DOI:** 10.1002/lom3.10182

**Published:** 2017-03-16

**Authors:** Nur Ili Hamizah Mustaffa, Maren Striebel, Oliver Wurl

**Affiliations:** ^1^ Institute for Chemistry and Biology of the Marine Environment, Carl Von Ossietzky Universität Oldenburg Wilhelmshaven Germany

## Abstract

We developed an effective fluorometric technique to quantify extracellular carbonic anhydrase (eCA) present in natural seawater samples. The technique includes the separation of eCA from cells to achieve low detection limits through high signal : noise ratios. eCA was efficiently extracted from cell membranes by treatment with 0.1 M phosphate buffer containing 2.5 M NaCl. The free eCA specifically forms a fluorescent complex with dansylamide, and the detection limit of the complex is below 0.1 nM. We applied the technique to samples from different culture solutions and natural seawater collected from the Baltic Sea. We observed eCA concentrations to be in the range of 0.10–0.67 nM in natural seawater. The data indicated that this technique is very sensitive, accurate, and feasible for routine and shipboard measurement of eCA from natural seawater. It is therefore an effective and rapid tool to investigate the carbon acquisition of phytoplankton both in mono culture as well natural communities.

Extracellular carbonic anhydrase (eCA) is a zinc‐containing enzyme that catalyzes the slow interconversion between ${\text{HCO}}_{3}^{-}$ and CO_2_ at the cell surface (Aizawa and Miyachi 1986). In seawater, phytoplankton can be CO_2_‐limited because dissolved inorganic carbon occurs mainly as ${\text{HCO}}_{3}^{-}$. Due to the inefficient conversion toward ${\text{HCO}}_{3}^{-}$, eCA is important for cellular CO_2_ acquisition in phytoplankton. The mechanism of inorganic carbon acquisition in marine phytoplankton is species dependent, regulated based on CO_2_ and/or ${\text{HCO}}_{3}^{-}$ concentrations (Nimer et al. 1997). eCA is found outside the plasmalemma, either in the periplasmic space or attached to the outer cell wall (Kimpel et al. 1983; Coleman and Grossman 1984). Husic and Quigley (1990) and van Hille et al. (2014) found that eCA bound either to the cell or plasma membrane through ionic interaction in various species of the genus chlorophyte and cyanobacteria. eCA is constitutive in most of the marine phytoplankton, whereas the expression was regulated by the concentration of inorganic carbon (Nimer et al. 1999; Elzenga et al. 2000). Previous studies have shown that the activity of eCA in marine phytoplankton depends on the availability of inorganic carbon, light and the pH (Moroney et al. 1985; Rigobello‐Masini et al. 2003, 2006). Expression of eCA increased at low partial pressure of CO_2_ (pCO_2_) and light availability, but essentially the expression was reduced at conditions with higher pCO_2_. In addition to the carbonate chemistry, the taxonomic composition and cell size of a phytoplankton community influences the level of eCA expression (Martin and Tortell 2006, 2008).

Reinfelder (2011) points out that the internal accumulation of inorganic carbon by eukaryotic marine phytoplankton is limited by the diffusion of CO_2_ through their cell membrane. As a response to this, these phytoplankton have developed a carbon‐concentrating mechanism (CCM) that includes eCA expression to increase the pCO_2_ outside the cell membrane and, therefore, to increase diffusion flux. Moreover, it has been suggested that the biophysical and biochemical characteristics of the CCM are variable within and among dominant groups (diatoms, dinoflagellates, and coccolithophores) of eukaryotic phytoplankton; however, the precise role of eCA in the CCM in these phytoplankton remains unclear. eCA plays a central role in the marine carbon cycle, including inorganic carbon acquisition and photosynthesis, and it would therefore be interesting to quantify it. In addition, the hypothical enrichment of eCA in the upper surface layer of the ocean, which was proposed for the first time 45 yr ago (Berger and Libby 1969), can potentially enhance the CO_2_ exchange rate. Matthew (1999) reported that CA may monitor the global air‐sea CO_2_ flux based on experiments conducted in a laboratory tank; however, experiments under natural conditions are not feasible due to the lack of analytical tools to detect the levels of naturally occurring CA. It is clear that eCA has the potential to increase the CO_2_ storage capacity of the ocean by decreasing the calcification rate and enhancing carbon overproduction (Riebesell 2004). Increase in the level of CO_2_ in the oceans, termed as ocean acidification, is expected to decrease the ${\text{HCO}}_{3}^{-}$ : CO_2_ ratio and seawater pH by approximately 65% and 0.35 units, respectively, by the end of this century (Wolf‐Gladrow et al. 1999). It has been suggested that ocean acidification can change the cellular mechanisms involved in the acquisition of inorganic carbon (Koch et al. 2013). Further, the role of eCA in the biogeochemical cycling of zinc, cadmium, and cobalt has also been discussed previously (Morel et al. 1994; Xu et al. 2008).

Despite the important role of eCA in marine biogeochemical cycles, there are no analytical techniques that can be used to assess its concentration or activity in natural seawater. Due to the instability of eCA in stored samples, a rapid and feasible technique is required for shipboard measurements. Spectrophotometric, radiotracer, membrane inlet mass spectrometry (MIMS), and isotope disequilibrium techniques have been applied for the indirect measurement of eCA in dense culture solutions. The electrometric technique based on pH differences (Wilbur and Anderson 1948) has been the method of choice to determine CA activity for many decades. However, this method displays a nonlinear response over the range of enzyme concentrations, and therefore has low accuracy and sensitivity. The radiotracer technique is rapid and has high sensitivity for measuring CA activity (Stemler 1993), but the handling and disposal of the required radioactive material is subject to strict safety regulations, which makes this technique unsuitable for routine measurements on board research vessels. The MIMS and isotope technique has also been used in several studies (Elzenga et al. 2000; Martin and Tortell 2006; Tortell et al. 2006). However, the high costs, technical requirements and time‐intensive sample treatments make its routine use difficult: for example, it would be difficult to monitor temporal and spatial changes in eCA expression with this method. Li and Ci (1995) developed a fluorometric technique to measure CA activity in human serum based on the formation of a fluorescent complex between CA and dansylamide (Chen and Kernohan 1967). Matthew (1999) tried to apply their technique to the measurement of CA concentration in seawater, but high background signals prevented the efficient detection of natural levels of eCA in seawater.

Numerous studies have focused on measuring the activity of eCA in concentrated culture solutions (Coleman and Grossman 1984; Rigobello‐Masini et al. 2003) or lake water (Berman‐Frank et al. 1994). However, according to Mercado and Gordillo (2011), the data obtained from lab‐based studies using phytoplankton cultures only provide information about the fundamental processes, and there is a need for a technique to measure eCA under natural conditions. Tortell et al. (2006) reported the total CA activity of 1.5 U mg protein^−1^ (the sum of the intra‐ and extracellular activity) in frozen seawater using the MIMS technique, but the stability of the samples was not assessed. The expression of eCA, produced in situ by phytoplankton, relies on primary productivity and the pCO_2_ level (Matthew 1999). In marine ecosystems, ambient pCO_2_ levels periodically vary and are often linked to diurnal and seasonal cycles (Borges et al. 2006; Huertas et al. 2006). The natural variability in the CO_2_ level plays a role in regulating the composition of phytoplankton communities, primary production as well as the biogeochemical cycling of carbon and bio‐limiting elements in the sea (Finkel et al. 2009). Given the limitations of lab‐based assessments, field studies in natural communities are important for obtaining further insight into eCA in the context of marine biogeochemistry. There is therefore a clear need for a rapid and feasible technique to determine eCA concentrations under natural conditions.

To address the need to quantify the CA present in seawater, we developed a fluorometric technique to measure eCA after the separation of eCA from cell membranes to reduce background interference. This fluorometric technique was chosen for its sensitivity, specificity and suitability for shipboard measurements. We tested it under different conditions of separation and quantification, using freshwater and marine phytoplankton cultured in the laboratory. We applied the new technique for onboard measurements of natural seawater samples from the Baltic Sea, and present the first depth profiles of eCA in the water column.

## Material and procedures

### Preparation of reagents and standard solutions

All chemicals were of analytical grade, and aqueous solutions were prepared with purified water from a water purifier system (Arius 611 VF; Sartorius, Germany). A stock solution of CA (1 × 10^−4^ M) was prepared every 3–4 d by dissolving bovine erythrocyte CA (lyophilized powder, ≥ 3.500 Wilbur‐Anderson Unit/mg protein; Sigma Aldrich, Germany) in 0.1 M phosphate buffer solution (pH 7.4). A stock solution of 5‐(dimethylamino)−1‐napthalenesulphonamide (DNSA) (Sigma Aldrich, Germany) was prepared every 3–4 d by dissolving 2.0 mg of DNSA in 10 mL of 0.1 M HCl. According to Chen and Kernohan (1967), those stock solutions are stable for a week. All stock solutions were maintained at 4°C. The solutions of 0.1 M of phosphate buffer (1 M solution [pH 7.4] from Sigma Aldrich, Germany) and 0.1 M phosphate buffer containing 2.5 M NaCl were prepared as needed. For electrometric experiments, 20 nM of Trizma base buffer (Sigma Aldrich, Germany) was prepared by dissolving 2.43 g in 100 mL purified water and the pH was adjusted to 8.3 using 2N sulfuric acid (Carl Roth, Germany). The buffer solution was stored at 4°C. CO_2_‐saturated water was prepared by bubbling ice‐cold and purified water with CO_2_ for at least 1 h on the day of the experiments (Wilbur and Anderson 1948).

### Phytoplankton culture in the laboratory

The new technique was tested on four species of phytoplankton: the freshwater species *Chlamydomonas reinhardtii* (*C. reinhartii*) and five marine species, namely, *Chlamydomonas* sp., *Nannochloropsis* sp., *Thalassiosira* sp., *Prorocentrum micans* (*P. micans*), *Cylindrotheca closterium* (*C. closterium*) and *Phaeactylum tricornutum* (*P. tricornutum*). All culture solutions were cultivated as triplicates in 100 mL of algal medium in a 250 mL cell culture flask. We used f/2 growth medium for the marine (Guillard 1975) and WC medium for the freshwater cultures (Guillard and Lorenzen 1972). The cultures were maintained in a climate chamber at a constant temperature of 18°C, light intensity of 90 μmol photons m^−2^ s^−1^, and a day/night cycle of 12/12 h. The initial algal culture solution, which was prepared from stock cultures cultivated under the above‐described conditions for months after isolation, was transferred into the cell culture bottles. Chlorophyll *a* (Chl *a*) concentrations were considered to represent the phytoplankton biomasses, and it was measured at regular intervals using a fluorometer (AquaFluor®, TURNER Designs with a Chl *a* in vivo channel). As the Chl *a* concentration per cell can differ between different species, we drew calibration curves so as to convert the Chl *a* concentrations into species‐specific biovolume or abundance values: the Chl *a* concentrations were considered as a measure of phytoplankton biovolume (and abundance) for five samples of different densities each. The samples were then examined under an inverted microscope, and the cell number was counted using a previously described method (Utermöhl 1958). Species‐specific cell volumes were calculated based on the shape of the cells according to Hillebrand et al. (1999). For the measurement of eCA, 25 mL of each phytoplankton solution was harvested and filtered through GF/F glass microfiber filters (25 mm diameter; Whatman, UK) once during the exponential and once during the stationary phase (Supporting Information Fig. S1).

### Seawater samples

Seawater samples were collected from the Baltic Sea during the summer of 2015 on board the R/V *Meteor*. Surface water samples from a depth of 1 m (*n* = 18) were collected using a 12‐V DC Teflon gear pump and polypropylene tubing from a small boat. Additional samples were collected by sampling the water column on 30^th^ July (profile 1: depth, 225 m) and 14^th^ August 2015 (profile 2: depth, 80 m) using a conductivity–temperature–depth rosette sampler. After collection, the seawater samples were temporarily stored (within 30 min) at 4°C in high‐density polypropylene bottles before filtration and analysis. The sampling equipment was pre‐cleaned with 0.1% HCl and rinsed with deionized water before use.

### Chl *a* measurement in seawater

The Chl *a* concentrations of seawater (1 m depth) were measured using an acidification fluorescence technique (Lorenzen 1967). The fluorometer (Jenway 6285; Bibby Scientific Ltd., UK) was calibrated before measurements were taken by using pure Chl *a* extract from spinach (Sigma Aldrich, Germany). Initially, 500 mL of the samples were filtered under vacuum on GF/F filters (Whatman, UK) and stored at −80°C until analysis in a land‐based laboratory. Samples were stored for 4 weeks. Filtered samples were extracted in 8 mL of 90% acetone for 24 h (at 4°C and in darkness). The relative fluorescence intensity (RFU) of the samples was measured before and after addition of 10% HCl at an excitation wavelength of 430 nm and an emission wavelength of 680 nm within 48 h. The Chl *a* concentration was calculated according to the method proposed by Lorenzen (1967).

### Filtration and extraction

For the measurement of eCA, 25 mL culture solutions were harvested and filtered on GF/F glass microfiber filters (25 mm diameter; Whatman, UK) once during the exponential and once during the stationary phase (see Supporting Information Fig. S1 for details). The filters were transferred immediately into a 1.5 mL microcentrifuge tube and maintained at −20°C until analysis. The filters were stored for up to 24 h. During field work, 200 mL of seawater was filtered immediately on GF/F glass microfiber filters (25 mm diameter; Whatman, UK). The filters were stored in a 1.5 mL microcentrifuge tube (Eppendorf) at −20°C and extracted within 24 h after filtration.

eCA was extracted from the cell surface using the salt‐induction dissociation technique described by Husic and Quigley (1990). First, the filters were suspended in ice‐cold pure water and centrifuged at 2000 × *g* for 5 min using a micro‐centrifuge (Carl Roth, Germany). The water was then removed using a micropipette, and the filters were again washed with ice‐cold pure water. The remaining pellets were suspended in ice‐cold 0.1 M phosphate buffer and centrifuged at 2000 × *g* for 5 min, and the liquid phase was carefully removed with a micropipette without disturbing the suspended pellets. The remaining pellets were treated with ice‐cold 0.1 M phosphate buffer containing 2.5 M NaCl. They were kept at 4°C for 10 min, after which the extracts were centrifuged at 2000 × *g* for 2 min. The final aliquots were collected carefully in microcentrifuge tubes.

### Development of fluorometric assay

The fluorescence spectra were obtained using a LS‐55 Fluorescence spectrometer (Perkin‐Elmer, U.S.A.) with excitation slits of 5 nm and a scanning rate of 1000 nm min^−1^. The excitation wavelength of DNSA‐CA complex was scanned between 260 nm and 300 nm, and the emission wavelength ranged between 400 nm and 500 nm. To validate specific binding of DNSA to CA, we tested the effect of high temperature upon fluorescence enhancement of the DNSA‐CA complex. Heat denaturation was carried out using a block heater (Thermo Scientific, Germany) at 70°C for 30 min. Fluorescence enhancement of DNSA‐CA complexes were measured before and after heating. For further tests on the specific binding, we selected five trace metals (i.e., zinc, cadmium, cobalt, lead, and iron) at concentrations of 2 nM to investigate their potential to interfere with the complexation of DNSA and CA. During the measurements, standard solutions of the trace metals were spiked subsequently into 2.5 mL of 0.1 M phosphate buffer containing 0.1 μM DNSA and 1.25 nM CA. With an excitation wavelength of 280 nm, we measured the emission wavelength between 400 nm and 500 nm after each addition of different trace metal solution.

### Extracellular carbonic anhydrase measurement

**Figure 1:**
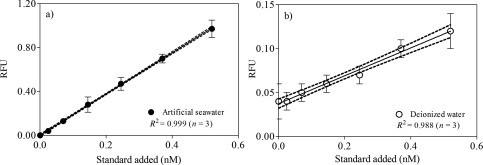
Curves of standard additions plotted against RFU values for (**a**) artificial seawater and (**b**) deionized water. The dotted lines represent 95% confidence bands.

The method for the determination of eCA concentration in this study was based on the binding of CA with dansylamide (DNSA) (Chen and Kernohan 1967; Li and Ci 1995). DNSA forms a highly specific fluorescent complex with CA by binding to its zinc atom at the active site of the enzyme, as a result of which the fluorescence intensity is enhanced (Chen and Kernohan 1967). The standard addition technique was applied to reduce matrix effects in the determination of eCA concentrations. Before measurement, the aliquots were diluted (1 : 20) with 0.1 M phosphate buffer to reduce the potential influence of chlorine ions in the formation of the DNSA‐CA complex. In the cuvette, 12.5 μL of DNSA was added to 750 μL diluted extracts; this was followed by addition of the CA stock solution. The fluorescence intensity of the DNSA‐CA complex was measured immediately with a standard filter fluorimeter (Jenway 6285; Bibby Scientific Ltd., Staffs, UK) at an excitation wavelength of 280 nm and an emission wavelength of 460 nm adapted from previous studies (Li and Ci 1995, 1998). The RFU of the samples was corrected by measurement of blank solutions containing only 0.1 M phosphate buffer and DNSA to eliminate background signals from free dansylamide. The RFU values were plotted against the standard additions, and the eCA concentrations were estimated by extrapolating the line to the *x*‐axis (Fig. 1). All measurements were conducted in triplicate.

### Electrometric technique

For method comparison, we measured eCA activity using the electrometric technique (Wilbur and Anderson 1948; van Hille et al. 2014). Briefly, the method is based on the time period for the pH to drop from 8.3 to 6.3 after the addition of CO_2_‐saturated water. CA was suspended in 3.0 mL 20 nM ice‐cold Trizma base buffer (pH 8.3). The time required for the pH to drop from 8.3 to 6.3 was measured in the absence (*t*_0_) and in the presence of CA in a range of 1.25–6.3 nM (*t*) after the addition of ice‐cold CO_2_‐saturated water. The temperature‐compensated pH was monitored with a pH meter PCD 650 using a glass electrode (Eutech Intruments, Thermo Scientific, Germany). The experiment was performed in a closed system at a temperature ranging between 0°C and 3°C. The activity of eCA was expressed in Wilbur‐Anderson Units (WAU) using the following formula:

(1) $$\text{WAU}\;=\;\left(t_{0}/t\right)\;-\;1$$

CA stock solution with known activity between 1.26 WAU and 6.39 WAU were prepared and measured in triplicates to validate our new fluorometric technique.

### Statistical analysis

The statistical analysis was performed using GraphPad PRISM 5.0. Differences in the concentration of eCA between phytoplankton species and between growths stages were considered to be significant at *p* ≤ 0.05, with a 95% confidence level. Spearman's correlation test was used to determine the correlation between normalization of eCA concentration with Chl *a* and normalization of eCA with biovolume. The results are presented as average ± standard deviation (SD), unless otherwise indicated.

## Assessment

### Sample storage

**Figure 2:**
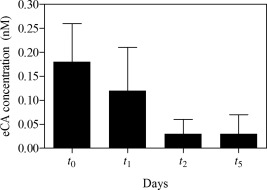
eCA concentration vs. storage time for filtered *C. reinhardtii* samples (*n* = 3 for each day).

The development of a storage protocol was essential to minimize loss of the analyte eCA. The optimal storage time of the filtered samples was determined using *C. reinhardtii* samples (*n* = 12) for 5 d. The first batch of filtered samples (*n* = 3) was extracted and analyzed immediately (*t*_0_), while other filtered samples were maintained at −20°C and analyzed on the following days (*t*_1_, *t*_2_, and *t*_5_). Generally, immediate extraction and measurement (*t*_0_) produced more reliable results (Fig. 2). One day of storage (*t*_1_) caused the mean concentration of eCA to decrease to 67%, but the difference between the concentration at *t*_1_ and *t*_0_ was not significant (*p* = 0.4368, *t*‐test). The eCA concentrations decreased continuously up to 6‐fold on the following days (*t*_2_ and *t*_5_); this probably included losses that occur during freezing and thawing. Therefore, immediate filtration, extraction, and measurement are recommended to ensure the highest level of accuracy.

### Potential interferences on the DNSA‐CA complexation

**Figure 3:**
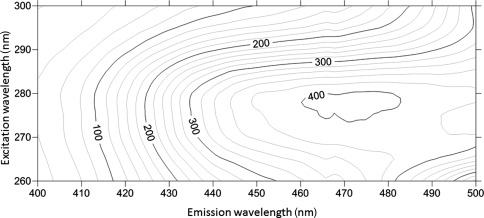
fluorescence spectra for DNSA‐CA complex in 0.1 M phosphate buffer solution. Contours represent as fluorescence intensity.

**Figure 4:**
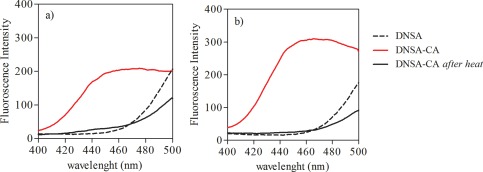
Heat denaturation effect on emission peak of DNSA‐CA complex in (**a**) phosphate buffer and (**b**) *Chlamydomonas* sp.

**Figure 5:**
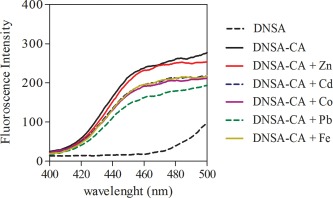
Potential interference of trace metals (i.e., zinc, cadmium, cobalt, lead, and iron) on DNSA‐CA complex in 0.1 M phosphate buffer. Trace metal solutions were added to DNSA‐CA complex accordingly.

Husic and Hsieh (1993) reported that the binding of CA to dansylamide resulted in a maxima of the fluorescence intensity at an emission wavelength of 468 nm by using an excitation wavelength of 280 nm or 326 nm. Using 0.1 M phosphate buffer solution, we observed that DNSA‐CA complex have maxima at an emission wavelength of 460 nm using an excitation wavelength of 280 nm (Fig. 3). However, the enhancement in the fluorescence intensity can potentially origin from the binding of DNSA with other molecules than CA. Therefore, we tested the fluorescence intensity of the DNSA‐CA complex before and after heat denaturation. Indeed, we observed reduction of the fluorescence signal after heat treatment at 70°C for 30 min. The fluorescence intensities were reduced by 83% and 91% in CA standards (Fig. 4a) and extracts of *Chlamydomonas* sp. (Fig. 4b), respectively. DNSA binds directly to the zinc atom embedded in the CA molecule (Chen and Kernohan 1967). For this reason, we tested potential interferences caused by trace metals (i.e., zinc, cobalt, copper, cadmium, lead, and iron) on the fluorescence intensity of the DNSA‐CA complex. The addition of the trace metals caused no increase in the fluorescence intensity (Fig. 5). The observed slight decrease of the intensity was probably caused by the time required for the successive measurements (e.g., 15 min) and instability of the initial DNSA‐CA complex.

### Linearity of the standard addition technique

We compared the calibration curves using artificial seawater and deionized water by correlating the background‐corrected RFU with additional standard concentrations (Fig. 1). We observed a general linear response to a range of standard additions (between 0.25 nM and 0.60 nM CA) in both media. However, we noted that the calibration curve for artificial seawater (Fig. 1a) showed more consistent fluorescence intensity in response to a range of standard additions, as demonstrated by the 95% confidence intervals of the regression lines. The averaged regression coefficient (*R* ^2^) values for artificial seawater and deionized water were 0.999 and 0.998, respectively. The slopes of the standard curves (*n* = 3), that is, the fluorescence intensity in response to increase in the CA concentration, in artificial seawater and deionized water were 1.879 ± 0.015 and 0.159 ± 0.008, respectively. Matthew (1999) suggested that seawater can serve as a good medium for formation of the DNSA‐CA complex. Thus, DNSA‐CA complexation may be suitable for determining the eCA concentration in natural seawater. We suggest using artificial seawater for preparing calibration solutions.

### Detection limit

The limit of detection was calculated as the average value of ten blank measurements plus three times its standard deviation, as outlined by Wurl (2009). Ten replicates of artificial seawater (*n* = 10), referred to blank samples, were filtered on GF/F glass microfiber filter, extracted, and measured using the same methods as those for the real samples. RFU values were plotted against standard additions, and blank concentrations were estimated based on the *x*‐axis intercept. The average blank value was 0.05 ± 0.01 nM. The limit of detection of eCA in seawater was 0.09 nM (*n* = 10), with a relative standard deviation of 26%.

### Effect of buffer salinity on extraction efficiency

**Figure 6:**
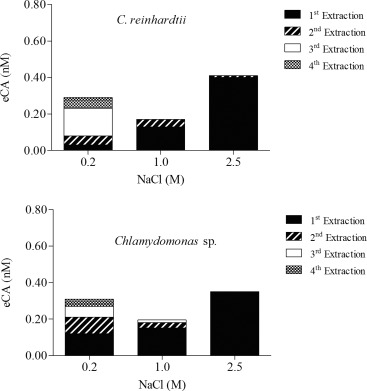
Efficiency of eCA extraction from cells using different NaCl concentrations.

Husic and Quigley (1990) reported that treatment with a saline solution can break the ionic bond between eCA and the cell wall. We examined the efficiency of different salt concentrations with the aim of separating eCA from the cell wall and, therefore, reducing noise and backscattering during fluorometric detection, which has been observed by Matthew (1999). We extracted eCA from *C. reinhardtii* and *Chlamydomonas* sp. using 0.2 M, 1.0 M, and 2.5 M of NaCl in 0.1 M phosphate buffer (pH 7.4). Figure 6 shows the relationship between eCA concentrations and the molar concentrations of NaCl used for extraction. Our observations suggest that eCA extracted from both *Chlamydomonas* species increased with the salt content of the extraction medium. When the cells were treated with 0.2 M NaCl, only 10–39% of the eCA was extracted from both freshwater and marine cells during the first step of extraction. The extracted concentrations of eCA increased as the NaCl concentration increased and reached a maximum with 2.5 M NaCl. Further extraction steps with 2.5 M NaCl did not result in an increase in the eCA concentration, which indicated that the extraction was complete with a single treatment. Overall, treatment with the extraction medium containing 2.5 M NaCl showed satisfactory extraction efficiency with a single step, as shown in Fig. 6.

### Interference by free chlorine ions

**Figure 7:**
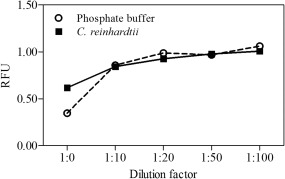
Dilution factors to reduce interference by free Cl^–^.

Li and Ci (1995) reported that a 1200‐fold molar excess of NaCl has no effect on the fluorescence intensity of the DNSA‐CA complex. However, we used high concentrations of NaCl, with a 250 × 10^6^ fold molar excess, during our extraction process, which led to interference by free Cl^–^. In order to minimize the interference, five series of dilutions with phosphate buffer were tested before fluorometric measurement (Fig. 7). First, a standard solution was diluted 1 : 20 with phosphate buffer; this dramatically enhanced the fluorescence intensity by up to 61%. A similar trend was observed with *C. reinhardtii* extracts. Thus, higher dilution factors do not increase the fluorescence intensity. Our observation shows that a dilution factor of 1 : 20 reduces interference by Cl^–^ and keeps the detection limit at a level suitable for the quantification of eCA in natural seawater.

### Comparison with electrometric method

**Figure 8:**
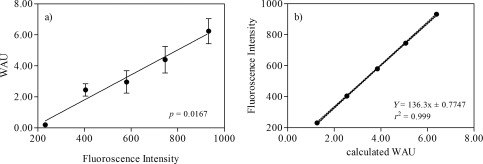
(**a**) Correlation between fluroscence intensity of CA standard solution and measured eCA activities (expressed in Wilbur‐Anderson Unit) using electrometric method, (**b**) linear regression of calculated eCA activities correspond to their fluoroscence intensity.

The comparison of our technique with the classical electrometric (Wilbur and Anderson 1948), or so‐called pH drift assay, showed a significant correlation (Spearman, *p* < 0.0167) between the outputs of WAU and eCA concentration (Fig. 8a). It highlights the validity of our technique. Furthermore, the measured fluorescence intensity were linear related to the known activity of the standards (*r* ^2^ = 0.999) (Fig. 8b) meaning that measured CA concentrations can be associated to activities, i.e., for comparison with earlier studies using the electrometric, MIMS or isotopic disequilibrium techniques. However, different CA isoforms may have different activities (Silverman 1991) likely to cause diversions from the observed linearity.

### eCA concentrations in cultured phytoplankton solutions

**Figure 9:**
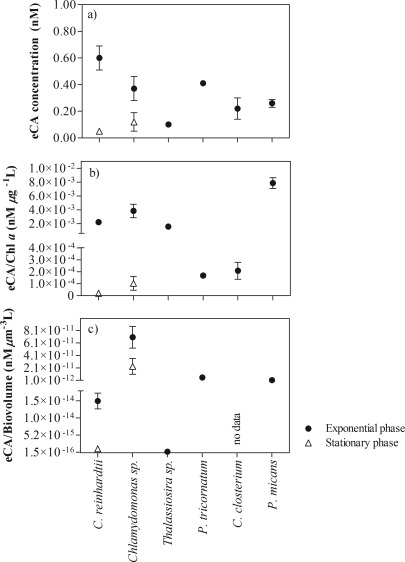
Concentration of eCA extracted from *C. reinhardtii*, *Chlamydomonas* sp., *Thalassiosira* sp., *P. tricornutum*, *P. micans*, and *C. closterium* during the exponential and stationary growth phases. The concentrations of eCA are shown as (**a**) absolute concentrations, (**b**) concentrations of eCA per μg Chl *a* per L and (**c**) concentrations of eCA per unit of phytoplankton biovolume (μm^3^•L^−1^). Values represent the means of triplicate cultures, and error bars represent the standard deviation (± SD).

The concentrations of eCA in freshwater and marine phytoplankton were determined based on the Chl *a* concentration and cell biovolume (Fig. 9). The cells were harvested during the exponential (day 3) and stationary phase (day 24). During the exponential phase, the eCA concentration in freshwater *C. reinhardtii* (0.60 ± 0.09 nM, *n* = 3) was higher than that in the marine species *Chlamydomonas* sp. (0.37 ± 0.09 nM, *n* = 3) at a moderate level of significance (*p* = 0.0372, *t*‐test). We detected low concentrations of eCA in *Thalassiosira* sp. during the exponential growth phase (0.10 ± 0.02 nM, *n* = 3). eCA was not detectable in *Nannochloropsis* sp. during both growth phases, as reported by Elzenga et al. (2000). Nimer et al. (1997) reported that *Nannochloropsis* sp. do not express eCA; therefore useful as negative control for testing the validity of our technique. Additionally, we tested our technique to other marine taxa such as dinoflagellate (*P. micans*) and diatoms (*P. tricornatum* and *C. closterium*), and the cells were harvested during exponential growth phase. eCA concentration in *P. tricornatum* (0.41 ± 0.02 nM, *n* = 3) was significantly higher than in *P. micans* (0.26 ± 0.02 nM, *n* = 3) and *C. closterium* (0.22 ± 0.08 nM n = 3) (*p <* 0.05, one way ANOVA, Tukey's multiple comparison test). It suggested that our technique is applicable to a wide range of taxa.

The concentration of eCA in the freshwater species *C. reinhardtii* decreased 12‐fold after the stationary growth phase was reached (0.05 ± 0.02 nM, *n* = 3) (Fig. 9a). The decrease was significant compared to the exponential phase (*p* = 0.0088, *t*‐test). On the contrary, no significant difference in the *Chlamydomona*s sp. (*p* = 0.1103, *t*‐test) between the exponential and stationary phase. Interestingly, the difference between the two species during the stationary phase was insignificant (*p* = 0.1238, *t*‐test), which indicates that some species alter their carbon acquisition strategy (such as *C. reinhardtii* in our study), while others do not (*Chlamydomona*s sp.) The decreasing trend in eCA was observed even after normalization of the eCA concentrations with Chl *a* concentrations (Fig. 9b) or biovolume (Fig. 9c). We found a significant correlation between eCA concentrations per Chl *a* and per unit biovolume for *C. reinhardtii* (*r* = 0.998, *p* < 0.0001) and *Chlamydomonas* sp. (*r* = 0.958, *p* = 0.0025). The presence of higher concentrations during the exponential growth phase indicated the significant role of eCA in carbon acquisition for photosynthesis. These findings indicate the usefulness of the described technique in investigating the physiological role of eCA.

### eCA concentrations in seawater

**Figure 10:**
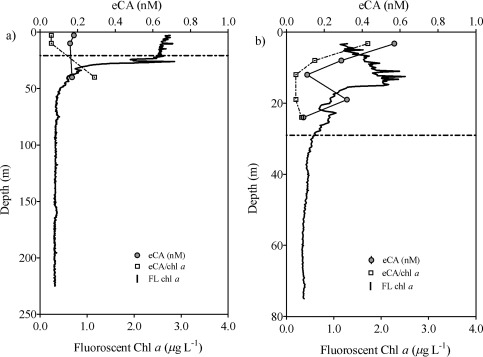
Depth profiles of eCA concentrations, fluorescent Chl *a* (FL Chl *a*) and the eCA/FL Chl *a* ratio in samples collected from the Baltic Sea. (**a**) Profile 1 (30^th^ July 2015) and (**b**) Profile 2 (14^th^ August 2015). The dotted line represents mixed layer depth.

The developed technique has been applied to the quantification of eCA in natural seawater samples. Table 1 shows the concentrations of eCA and Chl *a* from 1‐m deep seawater (*n* = 18). The average concentration of eCA and Chl *a* was 0.21 ± 0.15 nM and 6.82 ± 5.72 μg L^−1^, respectively. The normalization of the eCA concentration to the Chl *a* concentration ranged between 0.02 and 0.30 nM eCA μg Chl *a* L^−1^. Significant correlation was found between the eCA and Chl *a* concentrations (*r* = 0.6296, *p =* 0.0051) in marine surface water (1‐m depth). The vertical distribution of eCA and in situ fluorescence (calibrated according to Chl *a*) in the water columns are shown in Fig. 10, including the values at the mixed layer depth. In the first profile, eCA was detected from the upper water column (2.75 m) to a depth of 40 m, with an average concentration of 0.17 ± 0.01 nM. Intensive cyanobacterial blooms were observed at the locations of profile 1 (Fig. 9a). In the second profile (Fig. 9b), eCA concentrations were more variable, with an average of 0.32 ± 0.19 nM, even though the biomass values (fluorescence profiles) were similar. We detected the highest concentration of eCA at a depth of 3.75 m (profile 2, Fig. 10a), although the maximum fluorescence was detected at a depth of 10–15 m. Overall, our results indicate that the highest eCA concentrations are typically found in the upper 10 m of the water column, which is above the euphotic depth (i.e., 9 m for profile 1). However, due to the limited data set, we were unable to observe any meaningful correlations between the concentrations of eCA and Chl *a*.

**Table 1:** Concentrations of eCA and Chl *a* and the eCA/Chl *a* ratio in surface water at 1 m depth (*n* = 18).

	eCA (nM)	Chl *a* (μg L^−1^)	eCA/Chl *a* (nM μg^−1^ L)
*N*	18	18	18
Avg	0.22	5.86	0.07
SD	0.15	4.01	0.08
Min	0.10	0.90	0.02
Max	0.67	13.30	0.30
Median (25%)	0.13	1.65	0.02
Median (50%)	0.14	6.10	0.04
Median (75%)	0.32	8.40	0.09

Avg, average; SD, standard deviation; Min, minimum; Max, maximum.

## Discussion

The concentrations of eCA in natural communities and seawater are not known because there are currently no efficient analytical techniques for measuring them. Tortell et al. (2000) measured total CA expression in coastal Pacific phytoplankton assemblages using the potentiometric method, but due to its low sensitivity, the authors were unable to detect CA expression in half of their samples. By using the MIMS technique, Tortell et al. (2006) and Martin and Tortell (2006) measured total CA activity in seawater and phytoplankton samples collected from the subarctic Pacific and Bering Sea, respectively. The authors reported a lack of eCA activity in a majority of the samples (10 out of 15), and even when eCA activity was detected, the data were highly unclear. Matthew (1999) points out that CA is a large protein molecule whose activity depends on its relatively fragile macromolecular structure, and that its activity may be inhibited by protease enzymes produced by bacteria. Our technique showed that eCA is stable only for 24 h, so we suggest the lack of eCA activity reported in the previous studies may be related to CA degradation during prolonged storage, e.g., 3 months in the studies of Tortell et al. (2006) and Martin and Tortell (2008). Additionally, Rost et al. (2003) suggested that the MIMS technique has limitations for measurement of natural phytoplankton assemblages due to the presence of mixed autotrophic and heterotrophic communities, as a result of which the net CO_2_ and O_2_ fluxes may have been low. In an attempt to overcome these problems, we developed a simple and sensitive technique to quantify eCA concentrations at the nanomolar level from phytoplankton and seawater using a fluorometric technique. Owing to the simplicity of the technique, we were able to measure samples onboard within 24 h of their collection. Indeed, with our technique, it is possible to overcome the disadvantages of existing techniques that were mentioned previously. By using the filtration and extraction technique with an NaCl solution, we were able to minimize background fluorescence and multiple scattering problems, which are known to produce a poor signal : noise ratio in natural seawater, as reported by Matthew (1999). Consequently, our technique has a limit of detection of 0.09 nM. Moreover, we proved that our technique is able to quantify eCA concentrations at different CO_2_ conditions (see Supporting Information Fig. S2 for details).

Based on the fluorescence spectra of DNSA‐CA complex (Fig. 3), we observed an emission peak between 460 nm and 480 nm using an excitation wavelength of 280 nm. The wavelength of the maxima in the emission from our study correspond to wavelengths reported previously (Husic and Hsieh 1993; Li and Ci 1995). Free DNSA emits at 520 nm, but binding to the active site of the CA molecule, an efficient energy transfer occurs and caused a shift in the spectrum from 520 nm to 460 nm (Chen and Kernohan 1967). The disappearance of the emission peak (Fig. 4) after heat denaturation proved that fluorescence enhancement at 460 nm was only due to DNSA‐CA binding as heat treatment degraded CA. DNSA is very selective ligand which requires the presence of zinc or cobalt at the active site of CA to form a strong protein‐ligand binding (Chen and Kernohan 1967). Nair et al. (1996) suggested that binding with sulfonamide is most avid to the zinc holoenzyme compared to enzymes incorporating other metals. The network of hydrogen bonds around the sulfonamide group and the bonding between zinc and nitrogen are both crucial components of the interaction between DNSA and CA (Krishnamurthy et al. 2008). Coleman (1967) observed additional binding of DNSA with Co^2+^ and Cd^2+^ holoenzymes but Morel et al. (1994) suggested enzymes with substituted Co^2+^ and Cd^2+^ are less active than the native zinc form. Thompson et al. (1998) pointed out that substitution of metals ion for zinc decreased the affinity of the enzyme to bind with DNSA. Nevertheless, DNSA binds with other metals (Bargossi et al. 2000) but our results suggested that the additional binding has no influence at the fluorescence intensity at the emission wavelength of 460 nm (Fig. 5). For example, Kavallieratos et al. (2005) reports the binding of lead by DNSA, and an emission wavelength at 516 nm of the DNSA‐lead complex. Husic and Hsieh (1993) reported that non‐fluorescent sulphonamide and anions (i.e., Cl^–^) inhibit the CA activity and reduce fluorescence intensity. Therefore, we applied a dilution factor of 1 : 20 to reduce interference of free Cl^–^ with the DNSA‐CA complex. The steps of filtration, extraction, and centrifugation of our techniques remove the dissolved fractions of trace metals and organics reducing any potential of interferences to a minimum.

We applied this new technique to phytoplankton cultures, and the results showed that the expression of eCA was dependent on the species and growth phase. Freshwater *C. reinhardtii*, a species with high affinity for inorganic carbon, showed a significantly higher concentration of eCA than the other marine species examined in this study. Previously published data indicate that the expression of eCA in *C. reinhardtii* increases when the CO_2_ concentration is low (Coleman and Grossman 1984; Moroney et al. 2011). The abundant eCA activity observed in the freshwater species *C. reinhardtii* has previously been reported using a potentiometric method (Sültemeyer et al. 1993). However, due to the low sensitivity of the potentiometric method and lower expression of CA in marine phytoplankton, the potentiometric method is not appropriate for determining the concentration of eCA in marine species (Mercado et al. 1997). Nonetheless, failure to detect CA activity in marine phytoplankton with the potentiometric method does not necessarily mean that eCA has no physiological relevance (Elzenga et al. 2000). Nimer et al. (1994) used an electrometric method and reported that eCA activity was only detected when marine phytoplankton (*Emiliania huxleyi*) reached the stationary growth phase. However, eCA activity could not be detected in *E. huxleyi* using the isotope disequilibrium technique in both growing phases (Elzenga et al. 2000). On the contrary, the sensitivity of our technique was high enough to allow the detection of eCA from marine *Chlamydomonas* sp. in both growth phases: the concentration was found to be significantly lower than that in the freshwater species *C. reinhardtii*. Our technique allowed detection of eCA concentration from other marine taxa such as dinoflagellate (*P. micans*) and diatoms (*P. tricornatum* and *C.closterium*) during their exponential growth phase. Indeed, these species expressed eCA at the external cell surface to increase CO_2_ uptake rate for photosynthesis (Nimer et al. 1999; Hopkinson 2014). Burkhardt et al. (2001) aerated culture solutions of *P. tricornatum* and detected low eCA activity only in cultures adjusted to a low pCO_2_ level. Due to the increased concentrations of dissolved inorganic carbon, eCA activity remained undetectable at higher pCO_2_ levels as such conditions will reduce the expression eCA (John‐McKay and Colman 1997). Using potentiometric technique John‐McKay and Colman (1997) reported eCA activities from several strains of *P. tricornutum*.

We also demonstrated the efficiency to extract eCA from membranes with a saline solution from different taxa without any cells lyses being observed (see Supporting Information Figs. S3 and S4 for details). Indeed, salt dissociation technique has been established to break ionic interaction between eCA and the membrane from *C. reinhardtii* (Husic and Quigley 1990). van Hille et al. (2014) applied salt dissociation technique to the cyanobacterium *Spirulina platensis* and *Dunaliela salina*, a highly salt‐tolerant cell. van Hille et al. (2014) and our results from different culture solutions and natural communities support the efficiency of the salt dissociation technique to extract eCA from other taxa. The different levels of eCA in among those species provide compelling evidence for the variability of the carbon acquisition strategy in phytoplankton species. In addition, Hobson et al. (2001) suggested that ecological factors are important in variability of eCA activities. It is possible that eCA plays different physiological roles in marine and freshwater species, as mentioned by Roberts et al. (1997). For example, Rost et al. (2003) reported large variations in both the efficiency and regulation of carbon acquisition in three difference functional groups of phytoplankton (the diatom *Skeletonema costatum*, dinoflagellate *Phaeocystis globosa* and coccolithophorid *E. huxleyi*). Martin and Tortell (2006) suggested the taxonomic composition of phytoplankton communities may influence eCA expression. Moreover, Martin and Tortell (2008) reported variation in both ${\text{HCO}}_{3}^{-}$ transport and eCA expression across a range of diatoms species with different cell morphologies, but they were unable to explain the underlying mechanism. A recent study by Shen and Hopkinson (2015) suggested that eCA activity increased with cell size due to the higher demand for CO_2_ as larger cells were more prone to limitation for the carbon acquisition by diffusion.

We showed that the eCA concentrations decreased significantly in the stationary phase in the freshwater species *C. reinhardtii* (Fig. 9a). This may imply that some species alter their carbon acquisition strategies, while others do not, for example, *Chlamydomona*s sp. For *Thalassiosira* sp., we detected low concentrations of eCA during the exponential growth phase, which is consistent with low eCA activities reported by Trimborn et al. (2009). Previous studies by Elzenga et al. (2000) and Nimer et al. (1997) reported an absence of eCA activities in *Thalassiosira pseudonanna* (*T. pseudonanna*) using potentiometric technique. Nevertheless, we did not observe expression of eCA in *Nannochloropsis* sp. during both growth phases, which may mean that the species acquire inorganic carbon through the active transport of ${\text{HCO}}_{3}^{-}$ instead of extracellular conversion to CO_2_ and diffusion (Elzenga et al. 2000). Similarily, *Thalassiosira* sp. may acquire majority of inorganic carbon as ${\text{HCO}}_{3}^{-}$ across the membrane. Low expression of eCA in both freshwater and marine *Chlamydomonas* sp. during the stationary phase is probably the result of a limited carbon pump (Nimer et al. 1992) and diffusive CO_2_ uptake that occurs during inorganic carbon acquisition. Additionally, the difference in eCA concentration between the two growth phases may be related to nutrient availability, especially during the exponential phase. Lower eCA concentrations during the stationary phase can also be explained by high cell densities and therefore reduced light penetration in this phase, which limits eCA expression. Indeed, Rigobello‐Masini et al. (2003) reported that 50% of eCA activity was lost when *Tetraselmis gracilis* was transferred from light to dark conditions (5 h dark period); this indicates the importance of light for the expression of eCA.

We next examine the best approach to normalize the expression of eCA in natural samples. We found similar trends in eCA concentrations after normalization with Chl *a* concentrations or biovolume. In *C. reinhardtii* and *Chlamydomonas* sp., the eCA concentrations decreased from the exponential to the stationary growth phase by 12‐fold and 3‐fold respectively (Fig. 9a). Similarly, after normalization with the biovolume, the eCA concentrations decreased 13‐fold and 3‐fold in *C. reinhardtii* and *Chlamydomonas* sp. respectively (Fig. 9c). This result indicated that the quantification of biovolume is an appropriate approach for normalizing data from laboratory studies using culture solutions. However, quantification of biovolume is time‐consuming and, therefore, not practical for shipboard assessment of natural samples, as analysis of mixed communities requires taxonomical classification. Additionally, microscopic counting and quantification of cell volumes in mixed communities may be prone to errors (Felip and Catalan 2000). However, Felip and Catalan (2000) reported a relationship between Chl *a* and biovolume (*r* = 0.66) in Lake Redó, which indicates that Chl *a* could be a useful tool for estimating phytoplankton biomass in natural communities. The observed correlations between the eCA concentrations per Chl *a* molecule and per unit of biovolume (both for *C. reinhardtii* and *Chlamydomonas* sp.) indicate that normalization to Chl *a* concentrations is an appropriate approach for the assessment of natural communities.

To our knowledge, there are no other data in the literature on eCA concentrations in natural seawater. The concentration of zinc in seawater may be considered to represent the possible concentration of eCA, as discussed by Emerson (1995). Zinc is a cofactor of CA, and it has been suggested that 80% of the zinc present in diatoms is bound by this enzyme (Morel et al. 1994). Furthermore, Emerson (1995) pointed out that the number of moles of zinc and CA found in complex should be similar, considering the difference in their molecular weights. Bruland (1989) and Donat and Bruland (1990) measured the concentrations of complexed zinc in seawater and reported that it was 1.2 nM and 2 nM, respectively. Morel et al. (1994) hypothesized that the low levels of zinc in surface water may limit CO_2_ uptake and the growth rate of phytoplankton via eCA. Therefore, the low level of zinc in seawater may reflect the distribution of eCA in seawater. However, our results show that the vertical concentration profiles of eCA were not similar to the typical profiles of zinc (Bruland 1980; Kim et al. 2015). Perhaps more data points at more depths are required to identify any profile similarities, especially in the upper part of the water column. Higher concentrations of eCA were found above the mixed layer depth, which indicates the demand for inorganic carbon by marine phytoplankton for photosynthesis. Light availability, alkaline pH, and CO_2_ limitation within the upper water column probably enhanced the expression of eCA and hence catalyzed the interconversion between ${\text{HCO}}_{3}^{-}$ and CO_2_ (Nimer et al. 1997). Indeed, we found the highest eCA concentrations at 3.75 m in profile 2 (Fig. 10b). Intensive and visible cyanobacterial blooms, characterized by high in situ fluorescence, which represent the Chl *a* levels, in the upper water column (Fig. 10a) indicate that the source of eCA is in the upper part of profile 1. For example, Aizawa and Miyachi (1986) confirmed the expression of eCA in cyanobacteria. We suggest that eCA was not detectable below the lower euphotic depths due to limited availability of light and the subsequent inhibition of the expression of eCA. Previous field studies have shown that CA expression is correlated with the CO_2_ levels. For example, Berman‐Frank et al. (1994) and Tortell et al. (2006) demonstrated a correlation between CA and CO_2_ levels during annual blooms of the dinoflagellate *Peridinium gatunese* in Lake Kinneret and subarctic Pacific phytoplankton assemblages. We report the first set of data on eCA concentrations in seawater and its increased concentration in the upper water column, down to 10 m, which can potentially modify air‐sea CO_2_ exchange, as hypothesized by Berger and Libby (1969) and later by Emerson (1995). More recently, Calleja et al. (2005) reported the importance of phytoplankton communities close to the sea surface in the control of air‐sea CO_2_ disequilibria. Based on all these findings, we suggest that eCA could play a key role in these disequilibria. The new technique described here allows for detailed investigation of real‐time eCA concentrations in natural seawater samples and will be useful for further research in this direction.

## Conclusions

We have developed a sensitive, simple, and feasible technique for quantifying eCA in natural seawater. The technique is cost‐effective, has improved throughput, and is suitable for shipboard measurements. Using this technique, we found the eCA concentration in the Baltic Sea to be between 0.10 nM and 0.67 nM. The information available about the role of eCA in marine biogeochemistry, e.g., carbon acquisition, is mainly based on laboratory studies and in mono culture solutions. This is therefore the first set of data on eCA concentrations in natural communities. We were also able to detect differences in the expression of eCA between species and growth stages. We have described the first known technique for assessing eCA concentrations in natural phytoplankton communities and, therefore, a new tool for future studies to assess the role of eCA in the marine carbon cycle and ecology.

## Supporting information

Supporting Information
