# Supplementary material for: Extracellular carbonic anhydrase: Method development and its application to natural seawater
Source: Limnol Oceanogr Methods. 2017 Mar 16;15(5):503–17. doi: 10.1002/lom3.10182 (PMC6378606; doi:10.1002/lom3.10182)
Supplement: Supplementary file 1 — Supporting Information [file LOM3-15-503-s001.doc]

**Supplementary**


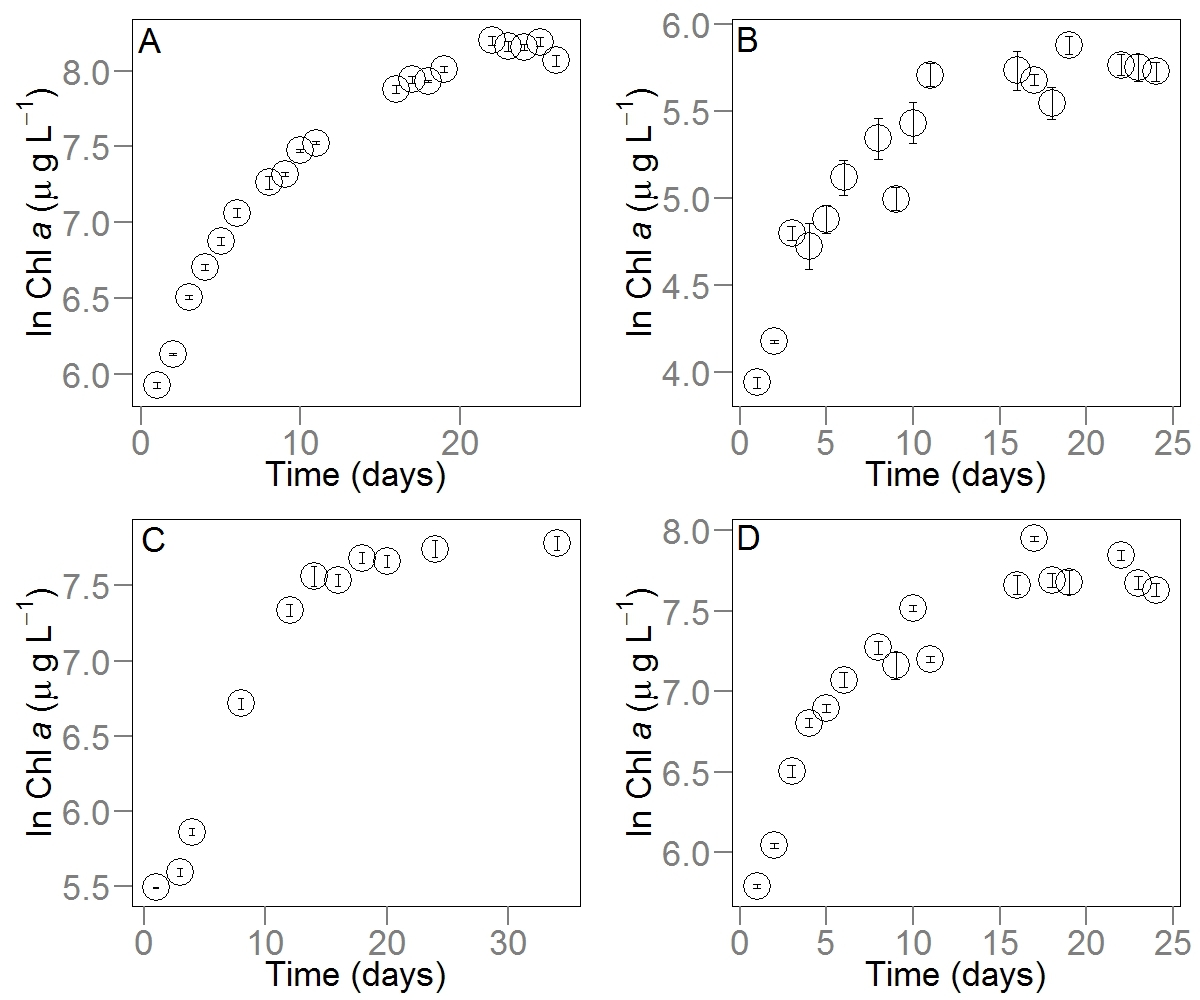


**Figure S1.** Biomass development of marine phytoplankton; (a) *Nannochloropsis* sp. (b) *Thalassiosira* sp., (c) *Chlamydomonas* sp. and (d) freshwater *C. reinhartii* based on Chl *a* concentrations (ln transformed).


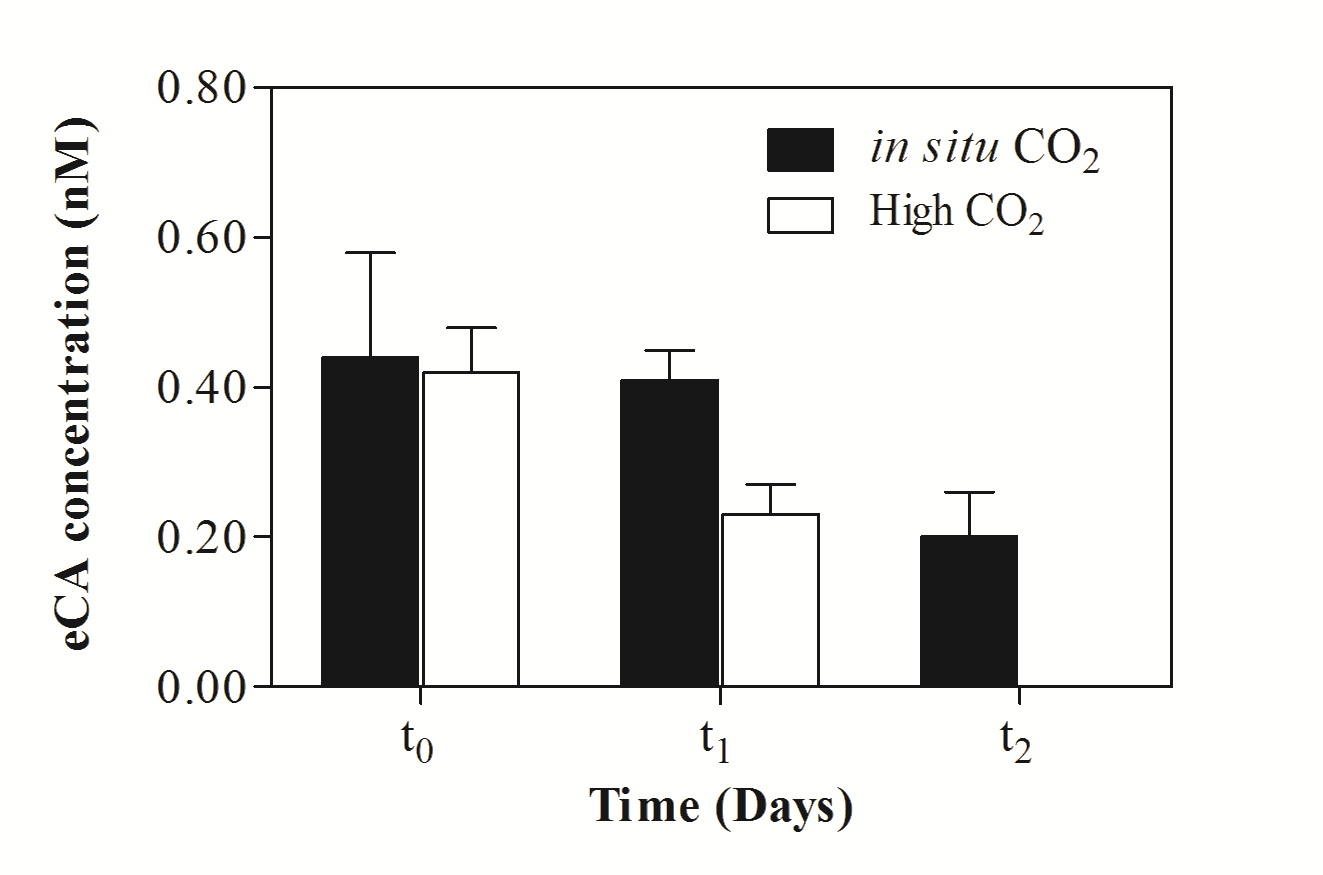


**Figure S2.** eCA concentrations at different CO_2_ conditions. Unfiltered sample from the depth with the maximum of chlorophyll *a* concentration (65 m depth) was collected using CTD cast at location -11.5125 N, 127.1523 E, on board *RV* Falkor (FK161010). Water was transferred immediately into 48 of 1 L shaded polycarbonate bottles (Nalgene) without headspace. For high CO_2_ condition, the seawaters were acidified by adding calculated volumes of HCl and HCO_3_^-^to achieve a level of 1300 µatm pCO_2_. Four replicate were used for each treatment. The bottles were placed in custom-build pool with a flow of ambient seawater. Sample were collected daily from day 1 (t_0_) until day 6 (t_5_) (20^th^ – 25^th^ October 2016). After t_2_ no eCA has been detected probably due to nutrient limitation and associated starvation.


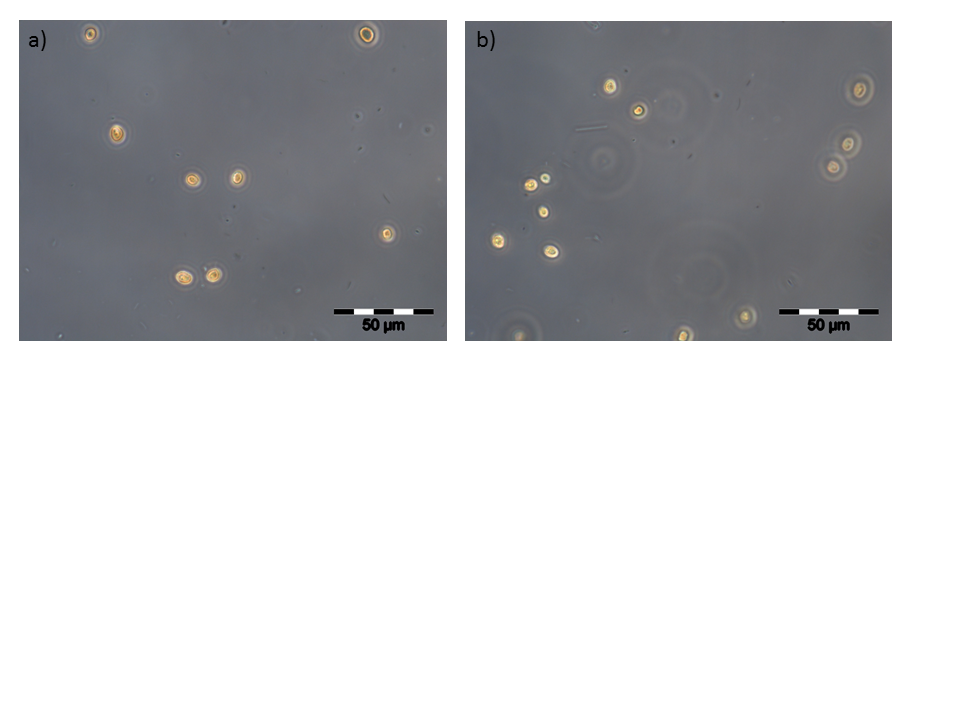


**Figure S3**. Microscopic images of (a) *Chlamydomona*s sp. from the culture solution. Image of (b) *Chlamydomona*s sp. after extraction with salt solution show no cell lysis. The cell was harvest during the exponential growth phase.


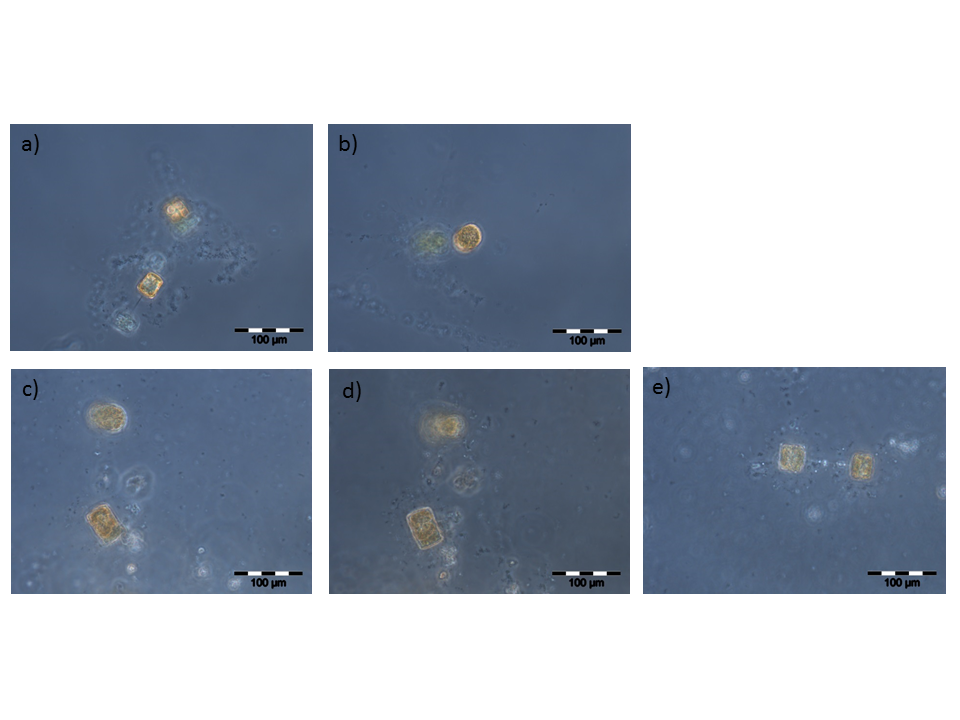
**Figure S4**. Microscopic images of (a) (b) *Thalassiosira* sp. before salt treatment. Images of *Thalassiosira* sp. after (c) 10 (d) 30 and (e) 60 minutes of adding salt solution. The cell was harvest during the exponential growth phase.
